# A comparison of machine learning algorithms and traditional regression-based statistical modeling for predicting hypertension incidence in a Canadian population

**DOI:** 10.1038/s41598-022-27264-x

**Published:** 2023-01-02

**Authors:** Mohammad Ziaul Islam Chowdhury, Alexander A. Leung, Robin L. Walker, Khokan C. Sikdar, Maeve O’Beirne, Hude Quan, Tanvir C. Turin

**Affiliations:** 1 Department of Community Health Sciences, University of Calgary, 3280 Hospital Drive NW, Calgary, AB T2N 4Z6 Canada; 2 Department of Family Medicine, University of Calgary, 3330 Hospital Drive NW, Calgary, AB T2N 4N1 Canada; 3 Present Address: Department of Psychiatry, University of Calgary, 3280 Hospital Drive NW, Calgary, AB T2N 4Z6 Canada; 4 Department of Medicine, University of Calgary, 3280 Hospital Drive NW, Calgary, AB T2N 4Z6 Canada; 5 Primary Health Care Integration Network, Primary Health Care, Alberta Health Services, Calgary, AB Canada; 6 Health Status Assessment, Surveillance and Reporting, Public Health Surveillance and Infrastructure, Provincial Population and Public Health, Alberta Health Services, 10101 Southport Rd. SW, Calgary, AB T2W 3N2 Canada

**Keywords:** Diseases, Medical research, Risk factors, Mathematics and computing

## Abstract

Risk prediction models are frequently used to identify individuals at risk of developing hypertension. This study evaluates different machine learning algorithms and compares their predictive performance with the conventional Cox proportional hazards (PH) model to predict hypertension incidence using survival data. This study analyzed 18,322 participants on 24 candidate features from the large Alberta’s Tomorrow Project (ATP) to develop different prediction models. To select the top features, we applied five feature selection methods, including two filter-based: a univariate Cox p-value and C-index; two embedded-based: random survival forest and least absolute shrinkage and selection operator (Lasso); and one constraint-based: the statistically equivalent signature (SES). Five machine learning algorithms were developed to predict hypertension incidence: penalized regression Ridge, Lasso, Elastic Net (EN), random survival forest (RSF), and gradient boosting (GB), along with the conventional Cox PH model. The predictive performance of the models was assessed using C-index. The performance of machine learning algorithms was observed, similar to the conventional Cox PH model. Average C-indexes were 0.78, 0.78, 0.78, 0.76, 0.76, and 0.77 for Ridge, Lasso, EN, RSF, GB and Cox PH, respectively. Important features associated with each model were also presented. Our study findings demonstrate little predictive performance difference between machine learning algorithms and the conventional Cox PH regression model in predicting hypertension incidence. In a moderate dataset with a reasonable number of features, conventional regression-based models perform similar to machine learning algorithms with good predictive accuracy.

## Introduction

Hypertension has long been documented as a substantial health burden that affects all population segments. Globally, hypertension causes 8.5 million of deaths every year and 7% of disease burden, making it one of the most significant risk factors for global mortality and disease burden^1–3^. Individuals with hypertension are at higher risk of developing life-changing and possibly life-threatening conditions^4^. One of the priorities of health and clinical research is to identify people at higher risk of developing an adverse health outcome, such as hypertension, so that they can be targeted for early preventative strategies and treatment^5^. Individuals at increased risk of developing hypertension could be recommended to change their lifestyle and behaviors (e.g., physical activity, dietary pattern, alcohol consumption, smoking, etc.) to reduce their risk. Prediction modeling can play a vital role in identifying high-risk individuals by estimating their risk of developing hypertension utilizing different underlying demographic and clinical characteristics called risk factors that are associated with hypertension^6–8^.

Various models have been developed that mathematically combine multiple risk factors to estimate the risk of hypertension in asymptomatic subjects in the population^9^. The regression-based methodologies, such as logistic regression and Cox regression, are the conventional approach for developing prediction models^10,11^. Machine learning algorithms recently emerged as a popular modeling approach that offers an alternative class of models with more computational flexibility^12^. Over the last few years, machine learning algorithms achieved significant success across a broad range of fields due to their superiority, such as their ability to model nonlinear relations and the accuracy of their overall predictions^13^. Nevertheless, the vast majority of existing hypertension risk prediction models are conventional regression-based models^14–23^. Machine learning-based models also exist in the hypertension prediction domain^24–35^. Machine learning algorithms sometimes struggle with reliable probabilistic estimation and interpretability^36,37^. Moreover, in clinical applications, machine learning algorithms often produce mixed results in predictive performance compared with conventional regression models^38–42^.

Data were primarily cross-sectional among the models where machine learning algorithms were used to predict hypertension^9^. Diagnostic models were built without considering or utilizing survival information where time is inherent in model building. Due to the lack of survival data utilization in predicting hypertension in the machine learning domain^9^, it is unclear how machine learning-based models will predict hypertension in survival data. A formal comparison in predictive performance between conventional regression-based hypertension prediction models and machine learning-based models in a survival setting is also absent^9^. There is also a scarcity of comparisons using the same dataset. This study investigated and compared five machine learning algorithms’ predictive performance with the conventional Cox PH regression model to predict the risk of developing hypertension in a survival setting.

## Methods

### Study population

This study used Alberta’s Tomorrow Project (ATP) cohort data, which is Alberta’s largest longitudinal population health cohort from the general population aged 35–69 years. ATP contains baseline and longitudinal information on socio-demographic characteristics, personal and family history of the disease, medication use, lifestyle and health behavior, environmental exposures, and physical measures. ATP has several questionnaires, and this study used data from 25,359 participants who completed the CORE questionnaire. A more detailed description of ATP data is provided in Supplementary Material (Appendix 1). In this study, eligible subjects were free of hypertension at baseline and consented to have their data linked with Alberta’s administrative health data (hospital discharge abstract data and physician claims data). Linking with administrative health data was completed to provide more comprehensive follow-up information on participants, necessary to determine hypertension incidence. We excluded 6,996 participants from the analysis who had hypertension at baseline and did not meet eligibility criteria. We also excluded 41 participants who responded to hypertension status questions at baseline as “don’t know” or “missing”. Eighteen thousand three hundred twenty-two participants remained after exclusion and were finally included in the analysis.

### Data pre-processing

To prepare the data for the machine learning algorithms, data pre-processing was performed. We began by evaluating the data's quality and consistency. Because our data originated from a single main source (ATP), we did not have any data quality issues such as mismatched data types (e.g., total family income in multiple currencies) or mixed data values (e.g., man vs. male). We examined the data for probable outliers. Our dataset had missing values on several candidate features ranging from 0 to 26%. As part of the data cleaning, missing values in the data set were imputed using multiple imputation by chained equations^43,44^. Multiple imputation, which entails making multiple predictions for each missing value, provides advantages over other approaches to missing data because analyses of multiply imputed data account for the uncertainty in the imputations and produce accurate standard errors^43,44^. One of the most prominent multiple imputation approaches is multiple imputation by chained equations (MICE), which is a realistic approach to constructing imputed datasets based on a collection of imputation models, one model for each variable with missing values. Since MICE uses a separate imputation model for each variable, it can accommodate a wide variety of variable types (for example, continuous, binary, unordered categorical, ordered categorical) and is therefore very flexible and can be used in a broad range of settings. Information on missing values for different candidate features is presented in the supplementary table (Table S1).

We used one-hot encoding, a standard strategy for dealing with categorical data in machine learning, in which a new binary feature is formed for each level of each category feature. When necessary, some of the categories of a categorical feature were merged or aggregated as part of data transformation to construct a new category of that categorical feature. The feature "ethnicity," for example, contains six subcategories: Aboriginal, Asian, White, Latin American Hispanic, Black, and other. The category "Asian" was developed by combining the categories South Asian, East Asian, Southeast Asian, Filipino, West Asian, and Arab. In addition, the levels of certain of the categorical features were occasionally combined to form a single binary feature indicating the presence or absence of the condition. For example, the feature "cardiovascular disease" was categorized as "yes" if any stroke, myocardial infarction, angina, arrhythmia, coronary heart disease, coronary artery disease, heart disease, or heart failure was present and as "no" if it was absent. For continuous features, we did not apply feature scaling techniques such as standardization or normalization in this study. Continuous features also remain continuous in the analysis.

### Selection of candidate features

We compiled a list of available potential candidate features before launching the analysis. We determined the possible candidate features for model development based on a literature search^9^, features used in the past^45^, and discussion with content experts. We initially considered 24 candidate features for the model development process. Given our model's intended clinical application, we did not consider any genetic risk factors/biomarkers as potential candidate features.

### Definition of outcome and features

The outcome of incident hypertension was determined through linked administrative health data using a coding algorithm. We used the relevant International Classification of Disease (ICD) 9^th^ and 10^th^ Version codes (ICD-9-CM codes: 401.x, 402.x, 403.x, 404.x, and 405.x; ICD-10-CA/CCI codes: I10.x, I11.x, I12.x, I13.x, and I15.x) and a validated hypertension case definition (two physician claims within two years or one hospital discharge for hypertension) to define hypertension incidence^46^.

The age of the study participants, body mass index (BMI), waist-hip ratio, diastolic blood pressure (DBP), systolic blood pressure (SBP), total physical activity time (total MET minutes/week), and total sitting time (the sum of the sitting times on weekdays and weekends) were all considered as continuous features. The remaining features were categorical. A detailed description of the features is provided in Supplementary Material (Appendix 2).

### Feature selection

Feature selection is a process where a subset of relevant features from a large amount of data is selected to filter the dataset down to the smallest possible subset of accurate features. It is imperative to identify the relevant features from a dataset and remove less significant features that contribute to the outcome to achieve better prediction model accuracy^10^. Feature selection methods can be classified into three categories: filter, wrapper, and embedded methods^47^. This study used two popular variants of filter methods in the survival analysis setting: a univariate Cox p-value and C-index^48^, two popular embedded methods of feature selection: RSF and Lasso, and a constraint-based method for feature selection: statistically equivalent signature (SES)^49^. More detail on these feature selection methods is provided in Supplementary Material (Appendix 3).

### Machine learning models

Modeling survival analysis (time-to-event data) requires specialized methods to handle unique challenges such as censoring, truncation, time-varying features, and effects. Censoring, where the event of interest is not observed due to time constraints or lost to follow-up during the study period, is challenging, and survival analysis provides different mechanisms to deal with such problems. Several machine learning algorithms have been developed and adapted to work with survival analysis data, effectively addressing complex challenges associated with survival data.

This study developed five well-known and popular machine learning algorithms, namely RSF, boosted gradient, penalized Lasso, penalized Ridge, and penalized EN. The machine learning algorithms chosen fall into three categories: penalized Cox regression (Lasso, ridge, and EN); boosted Cox regression (Cox model with gradient boosting); and random forests (RSF). A brief description of these models is provided in Supplementary Material (Appendix 4). The Cox PH model was included here as a conventional regression-based model (baseline) against which we compared the machine learning-based models.

### Feature importance

Feature importance is a tool that refers to a class of techniques for assigning scores to input features according to their usefulness in predicting a target feature. The relative scores can indicate which features are most relevant to the target and which are not. Feature importance helps interpret and explain machine learning algorithms by illustrating the predictive power of the dataset’s features. The goal of using feature importance in this study was to learn what features are important to different models so that we could interpret and discuss the model with others. Often, machine learning algorithms merely provide predictions and do not explain what elements contribute to their predictions or how their weights are calculated. This provides an interpretability challenge for machine learning algorithms, especially in clinical research, because readers are constantly interested in knowing the features that contribute to the prediction of a condition such as hypertension. Because machine learning techniques are hard to understand, we chose to show the importance of features in our work so that people could see which features helped predict hypertension.

There are a variety of methods for calculating the importance of a feature, and different modeling methodologies employ distinct methods for calculating feature importance metrics. The function for computing the importance of features in RSF, GB, and Cox PH models is based on Breiman’s permutation method^50^, where each feature is randomly permuted at a time, and the associated reduction in predictive performance is calculated. For the penalized models, the standardized regression coefficients’ magnitude was used to rank order the features according to their importance^51^. To ensure comparable rank-ordering across all models, the importance metrics’ absolute values for all the features were scaled to unit norm^52^.

### Statistical analysis

We first imputed the missing values. We then randomly split subjects into two sets: the training set, which included 67% (two-thirds) of the sample (*n* = 12,233), and the testing set, which included the remaining 33% (one-third) (*n* = 6,089). The two groups’ baseline characteristics were compared using the unpaired t-test or the *χ*^2^-test, as appropriate. We developed risk prediction models from the training data and assessed the models’ performance using the testing data. Five feature selection methods were employed to derive the most accurate risk prediction model for all the machine learning and conventional regression models. Features were first ranked according to their importance/scores/*p* values. Based on the features’ ranking, the top 20 features by each of the methods were selected. Due to the variations in the selected top 20 features by different methods, features that are common in all the methods are finally considered in model building.

Five machine learning algorithms and the conventional Cox PH model were developed in the training set. Machine learning algorithms have hyper-parameters that need to be selected to optimize model performance. We carried on tuning these hyper-parameters automatically within a tenfold nested cross-validation loop. Hyper-parameter values were chosen by applying 20 random iterations in the inner loop, and model performance was assessed in the outer loop. This ensured the repetition of model selection steps for each training and test data pair. The number of random variables for splitting and the minimal number of events in the terminal nodes were tuned when building the RSF. We fitted a Cox PH model as a base learner for GB models. The number of boosting iterations and the regression coefficients were tuned in GB. Parameter lambda was tuned for the penalized models, and the best value was chosen based on tenfold cross-validation. The models’ predictive performance was evaluated using the concordance index (C-index)^53^, which measures the proportion of pairs in which observation with higher survival time has a higher probability of survival as predicted by the model. The whole process was iterated ten times by sampling the original data with replacement.

**Figure 1:**
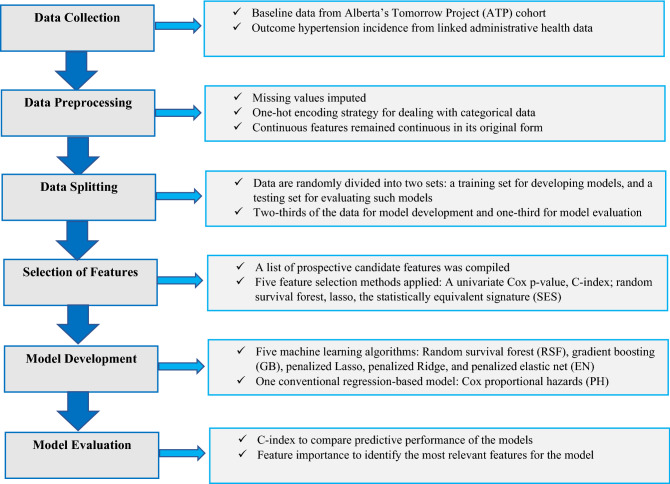
Graphical illustration of the workflow used for this study.

Moreover, the training data features were ranked according to their relative contribution to predicting hypertension incidence using various feature importance metrics. Graphical illustration of the workflow used for this study is presented in Fig. 1. The analyses were conducted using several packages^51,54–60^ of R software v 3.6.2. On reasonable request, the corresponding author may release the code for the analysis used in the current study.

### Ethics approval

The Conjoint Health Research Ethics Board (CHREB) at the University of Calgary granted ethical approval for this study (REB18-0162_REN2), and all methods were performed in accordance with the relevant guidelines and regulations. Informed consent was waived by the CHREB because the dataset used in this study consisted of de-identified secondary data released for research purposes.

### Consent to participate

The manuscript is based on the analysis of secondary de-identified data. Patients and the public were not involved in the development, design, conduct or reporting of the study.

## Results

We presented the baseline characteristics of the study participants in Table 1 and Supplementary Table S2. In Table 1, the study participants’ characteristics are compared according to the status of developing hypertension, while in Supplementary Table S2, characteristics are compared between training data and test data. During the median 5.8-year follow-up, 625 (3.41%) participants newly developed hypertension. In Table 1, most of the study characteristics were significantly different (*p* < 0.05) between those who developed hypertension and those who did not. These include age, sex, body mass index (BMI), waist-hip ratio (WHR), diastolic blood pressure (DBP), systolic blood pressure (SBP), total household income, highest education level completed, diabetes, cardiovascular disease, smoking status, working status, total sleep time, total sitting time, vegetable and fruit consumption, and job schedule. However, some study characteristics were not significantly different (*p* < 0.05), including marital status, residence, ethnicity, depression, family history of hypertension, alcohol consumption, total physical activity time, and physical activity. Overall, the study participants’ mean age was 50.99 years, and there were more females (*n* = 12,559, 68.55%) than males (*n* = 5,763, 31.45%). In Supplementary Table S2, no significant difference (*p* < 0.05) in study characteristics was observed between training and test data.

**Table 1:** Baseline characteristics of study participants according to the status of developing hypertension or not.

Variable	Categories	All participants (18,322)	Participants who developed hypertension (*n* = 625)	Participants who did not develop hypertension (*n* = 17,697)	*P* value
**Socio-demographic characteristics of groups**					
Age, years, mean (SE)		50.99 (0.07)	53.99 (0.35)	50.88 (0.07)	< 0.001
Sex, n (%)	Male (reference)	5763 (31.45)	250 (40)	5513 (31.15)	< 0.001
	Female	12,559 (68.55)	375 (60)	12,184 (68.85)	
Body mass index, kg/m2, mean (SE)		26.45 (0.04)	28.63 (0.21)	26.38 (0.04)	< 0.001
Waist hip ratio, mean (SE)		0.9093 (0.0006)	0.9363 (0.0033)	0.9085 (0.0006)	< 0.001
Diastolic blood pressure, mean (SE)		72.96 (0.08)	78.43 (0.47)	72.78 (0.08)	< 0.001
Systolic blood pressure, mean (SE)		119.71 (0.11)	132.36 (0.67)	119.40 (0.12)	< 0.001
Marital status, *n* (%)	Married and/or living with a partner (reference)	14,457 (78.91)	488 (78.08)	13,969 (78.94)	0.146
	Single, never married	1180 (6.44)	32 (5.12)	1148 (6.49)	
	Other (divorced, widowed, separated)	2685 (14.65)	105 (16.8)	2580 (14.57)	
Residence, *n* (%)	Urban (reference)	15,272 (83.35)	428 (68.48)	14,844 (83.88)	0.146
	Rural	3050 (16.65)	197 (31.52)	2853 (16.12)	
Total Household Income, *n* (%)	< $49,999 (reference)	2800 (15.28)	178 (28.56)	2627 (14.84)	< 0.001
	$50,000–$99,999	5912 (32.27)	229 (36.68)	5690 (32.15)	
	$100,000–$199,999	7174 (39.16)	177 (28.27)	6986 (39.48)	
	≥ $200,000	2436 (13.29)	41 (6.49)	2394 (13.52)	
Highest education level completed, *n* (%)	High school or below (none, elementary school, high school, trade, technical or vocational school, apprenticeship training or technical CEGEP) (reference)	6164 (33.64)	309 (49.35)	5854 (33.08)	< 0.001
	Diploma but below bachelor’s degree (diploma from a community college, pre-university CEGEP or non-university certificate, university certificate below bachelor’s level)	4926 (26.89)	163 (26.15)	4764 (26.92)	
	Bachelor’s degree or above (bachelor's degree, graduate degree (MSc, MBA, MD, PhD, etc.))	7232 (39.47)	153 (24.49)	7079 (40.0)	
Ethnicity, *n* (%)	Aboriginal	68 (0.37)	1 (0.16)	67 (0.38)	0.349
	Asian (South Asian, East Asian, Southeast Asian, Filipino, West Asian, Arab)	827 (4.51)	21 (3.4)	806 (4.55)	
	White (reference)	16,894 (92.21)	588 (94.03)	16,307 (92.14)	
	Latin American Hispanic	162 (0.89)	2 (0.32)	160 (0.9)	
	Black	97 (0.53)	2 (0.33)	95 (0.54)	
	Other (Jewish and others)	273 (1.49)	11 (1.76)	262 (1.48)	
Diabetes, *n* (%)		735 (4.01)	58 (9.28)	677 (3.83)	< 0.001
Cardiovascular Disease, *n* (%)		377 (2.06)	40 (6.4)	337 (1.9)	< 0.001
Depression, *n* (%)		2011 (10.98)	79 (12.64)	1932 (10.92)	0.179
Family history of hypertension, *n* (%)		10,946 (59.74)	396 (63.36)	10,550 (59.61)	0.061
Smoking Status, *n* (%)	Never (reference)	10,107 (55.16)	290 (46.37)	9823 (55.51)	< 0.001
	Former	6773 (36.97)	276 (44.15)	6491 (36.68)	
	Current	1442 (7.87)	59 (9.48)	1383 (7.81)	
Alcohol consumption, *n* (%)	Never (reference)	1279 (6.98)	56 (8.97)	1224 (6.92)	0.189
	≤ 1 time a week	9642 (52.63)	341 (54.52)	9307 (52.59)	
	2 to 3 times a week	3820 (20.85)	123 (19.77)	3689 (20.85)	
	4 to 5 times a week	1988 (10.85)	55 (8.74)	1938 (10.95)	
	≥ 6 times a week	1593 (8.69)	50 (8.0)	1539 (8.69)	
Working status, *n* (%)	Full time (reference)	11,449 (62.49)	352 (56.29)	11,057 (62.48)	< 0.001
	Part time	4596 (25.09)	182 (29.19)	4422 (24.99)	
	Other (looking after home, disable/sick, student, unpaid/voluntary)	1857 (10.13)	83 (13.23)	1803 (10.18)	
	Unemployed	420 (2.29)	8 (1.28)	415 (2.35)	
Total sleep time, *n* (%)	≤ 5 h (short sleep duration)	1192 (6.51)	47 (7.49)	1147 (6.48)	< 0.001
	6 h	3732 (20.37)	127 (20.33)	3604 (20.37)	
	7 h (reference)	7048 (38.46)	200 (32.02)	6847 (38.69)	
	8 h	5115 (27.92)	185 (29.66)	4929 (27.85)	
	≥ 9 h (long sleep duration)	1235 (6.74)	66 (10.49)	1170 (6.61)	
Total physical activity time, mean (SE)		3159.83 (21.43)	3183.97 (126.52)	3157.58 (21.68)	0.825
Total sitting time, mean (SE)		2488.53 (8.92)	2389.16 (49.14)	2490.98 (9.38)	0.043
Physical activity, *n* (%)	Low (first quartile of physical activity time and fourth quartile of sitting time) (reference)	1685 (9.19)	59 (9.47)	1678 (9.48)	0.707
	Moderate (second and third quartile of physical activity time and sitting time)	14,478 (79.02)	488 (78.12)	13,957 (78.87)	
	High (fourth quartile of physical activity and first quartile of sitting time)	2159 (11.78)	78 (12.40)	2062 (11.65)	
Vegetable and fruit consumption, n (%)	Low consumption (less than 5 servings of vegetable and fruit) (reference)	15,264 (83.31)	544 (87.05)	14,721 (83.18)	0.024
	Moderate consumption (less than 5 servings of vegetables but more than 5 servings of fruit OR more than 5 servings of vegetables but less than 5 servings of fruits)	2536 (13.84)	68 (10.84)	2469 (13.95)	
	High consumption (5 or more servings of vegetable and fruit)	522 (2.85)	13 (2.11)	507(2.87)	
Job schedule, n (%)	Regular daytime shift (reference)	12,866 (70.22)	385 (61.59)	12,452 (70.36)	< 0.001
	Other (evening shift, night shift, rotating shift, split shift, irregular shift, or on call)	5456 (29.78)	240 (38.41)	5245 (29.64)	

Table 2 presents feature rankings of all 24 candidate features, and Table 3 shows the top 20 features based on five different methods. Due to different methods' differences in the ranking, the top 20 selected features are not the same. We chose features common in the top 20 selected by different methods to avoid less relevant features in the model building process. Fourteen features were identified as common in all top 20 features and were included in the final model building process (Table 3, bold text). These included SBP, DBP, BMI, waist-hip ratio, diabetes, cardiovascular disease, age, job schedule, working status, total household income, residence, highest education level completed, family history of hypertension, and sex.

**Table 2:** Feature’s ranked based on five different approaches.

Feature	Ranking based on random survival forest relative importance	Ranking based on statistical equivalent signature	Ranking based on Harrel’s C-index/Somers’ Dxy rank correlation	Ranking based on Lasso Cox coefficients/variable importance	Ranking based on univariate Cox *p* values
Systolic blood pressure	1	1	1	13	1
Diastolic blood pressure	2	20	2	15	5
Body mass index	3	2	3	11	3
Waist-hip ratio	4	11	5	1	4
Diabetes	5	5	14	3	10
Cardiovascular disease	6	3	16	2	9
Age	7	4	4	14	2
Job schedule	8	6	6	4	7
Working Status	9	8	7	19	8
Total household income	10	7	9	6	6
Residence	11	13	10	5	12
Total sleep time	12	9	11	22	15
Highest education level completed	13	12	8	10	11
Family history of hypertension	14	17	18	12	16
Physical activity, quartiles	15	19	22	21	23
Smoking status	16	14	12	23	14
Total physical activity time	17	24	15	16	17
Depression,	18	21	21	9	24
Ethnicity	19	10	24	18	21
Sex	20	18	13	8	13
Total sitting time	21	22	23	17	22
Alcohol consumption	22	16	17	7	19
Marital status	23	15	20	24	20
Vegetable and fruit consumption	24	23	19	20	18

**Table 3:** The top 20 features selected by the different approaches with bold text indicates commonly selected features.

Random survival forest relative importance	Statistical equivalent signature	Harrel’s C-index/Somers’ Dxy rank correlation	Lasso Cox coefficients/variable importance feature	Univariate Cox p-values
**Top 20 features**				
**Systolic blood pressure**	**Systolic blood pressure**	**Systolic blood pressure**	**Waist-hip ratio**	**Systolic blood pressure**
**Diastolic blood pressure**	**Body mass index**	**Diastolic blood pressure**	**Cardiovascular disease**	**Age**
**Body mass index**	**Cardiovascular disease**	**Body mass index**	**Diabetes**	**Body mass index**
**Waist-hip ratio**	**Age**	**Age**	**Job schedule**	**Waist-hip ratio**
**Diabetes**	**Diabetes**	**Waist-hip ratio**	**Residence**	**Diastolic blood pressure**
**Cardiovascular disease**	**Job schedule**	**Job schedule**	**Total household income**	**Total household income**
**Age**	**Total household income**	**Working status**	Alcohol Consumption	**Job schedule**
**Job schedule**	**Working status**	**Highest education level completed**	**Sex**	**Working status**
**Working status**	Total sleep time	**Total household income**	Depression	**Cardiovascular disease**
**Total household income**	Ethnicity	**Residence**	**Highest education level completed**	**Diabetes**
**Residence**	**Waist-hip ratio**	Total sleep time	**Body mass index**	**Highest education level completed**
Total sleep time	**Highest education level completed**	Smoking status	**Family history of hypertension**	**Residence**
**Highest education level completed**	**Residence**	**Sex**	**Systolic blood pressure**	**Sex**
**Family history of hypertension**	Smoking status	**Diabetes**	**Age**	Smoking status
Physical activity, quartiles	Marital status	Total physical activity time	**Diastolic blood pressure**	Total sleep time
Smoking status	Alcohol consumption	**Cardiovascular disease**	Total physical activity time	**Family history of hypertension**
Total physical activity time	**Family history of hypertension**	Alcohol consumption	Total sitting time	Total physical activity time
Depression	**Sex**	**Family history of hypertension**	Ethnicity	Vegetable and fruit consumption
Ethnicity	Physical activity, quartiles	Vegetable and fruit consumption	**Working status**	Alcohol consumption
**Sex**	**Diastolic blood pressure**	Marital status	Vegetable and fruit consumption	Marital status

**Figure 2:**
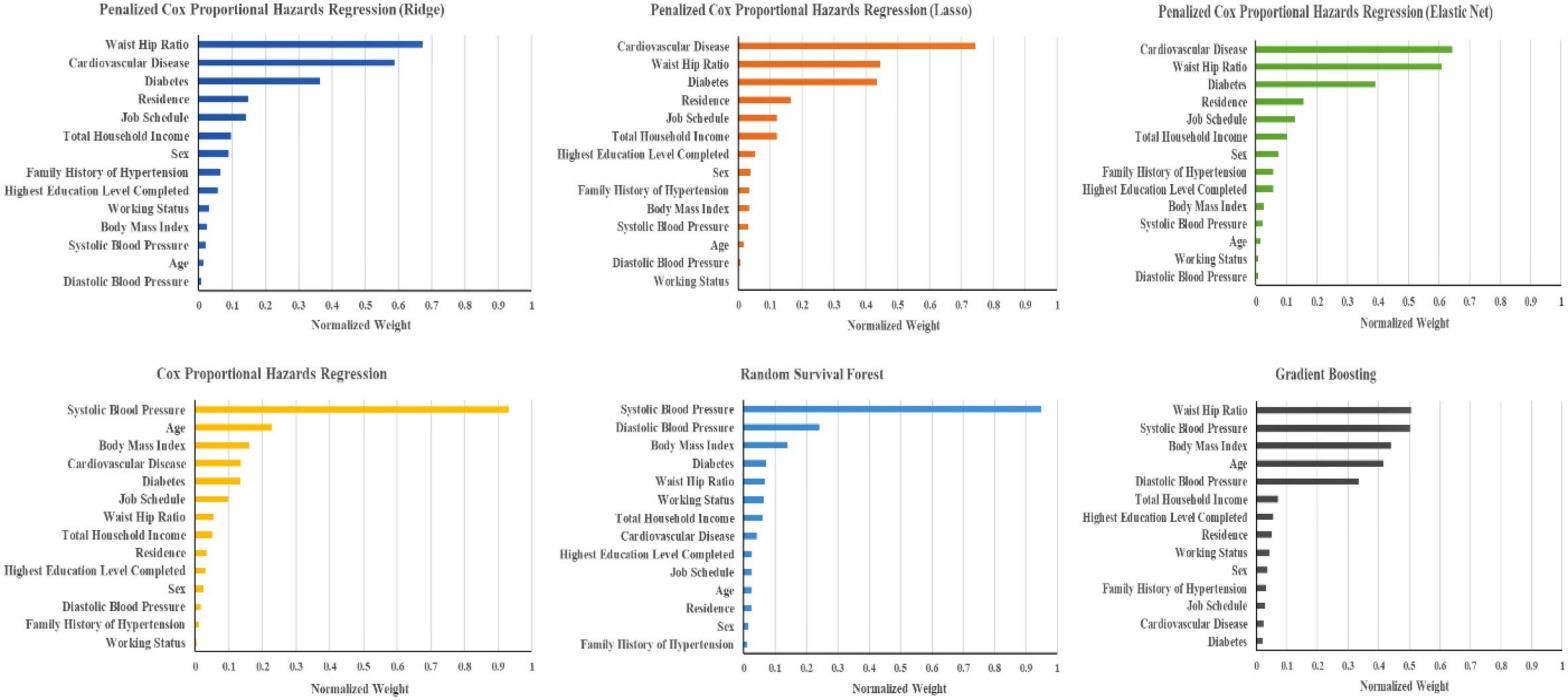
Features ranked according to their importance by the different model.

Figure 2 describes the relative importance of features concerning the prediction of hypertension incidence by six different model-building approaches. The waist-hip ratio was selected as the top feature by Ridge regression and GB. In contrast, cardiovascular disease was selected as the top feature by Lasso regression and EN regression. SBP was selected as the top feature by the Cox PH model and RSF. The waist-hip ratio, cardiovascular disease, diabetes, SBP, age, and BMI have been deemed the most important features considered by most modeling approaches. However, there are also variations in the rank ordering of important features across the investigated models.

**Figure 3:**
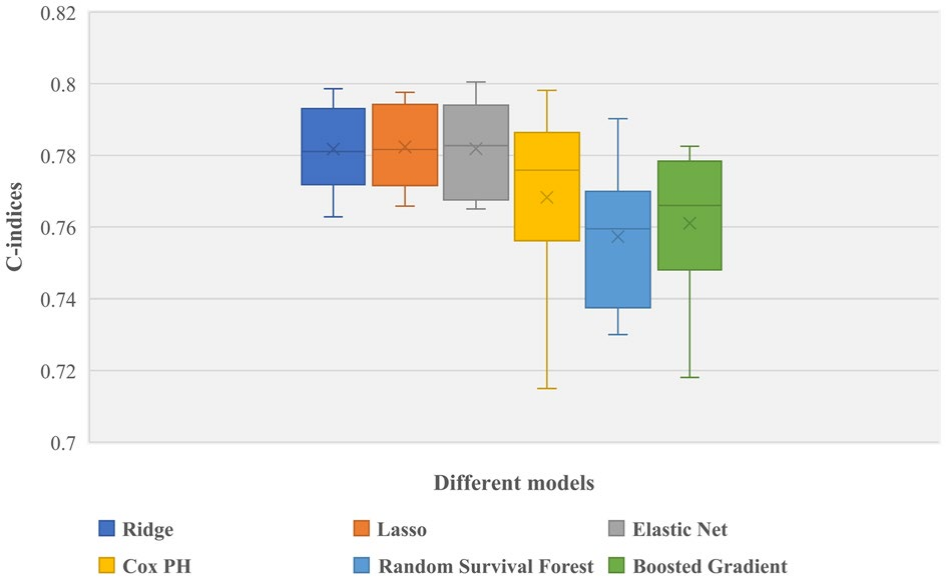
Boxplots showing the spread of values of the C-index produced by the different model.

Figure 3 describes the predictive accuracy of different models. There were negligible differences in the accuracy of machine learning and conventional regression-based Cox models. The average C-index for the machine learning algorithms Ridge, Lasso, EN, RSF, and GB was 0.78, 0.78, 0.78, 0.76, and 0.76, respectively. In comparison, the conventional regression-based Cox PH model’s average C-index was 0.77. Nevertheless, when penalized techniques were used, the models were a little better at making predictions.

## Discussion

This study examined the predictive accuracy of machine learning algorithms and compared their performance with the conventional regression-based Cox PH model to predict hypertension incidence. The predictive accuracy of the machine learning algorithms and the Cox PH model was good^61^, as the C-index was well over 0.70 in every case. Our findings suggest that the machine learning algorithm’s predictive accuracy is similar to the regression-based Cox PH model. These findings are consistent with our recent systematic review and meta-analysis, where no evidence of machine learning algorithms’ superior predictive performance over conventional regression-based models was observed^9^. According to our recent meta-analysis^9^, which is a pooled analysis of the papers included in our systematic review of hypertension risk prediction models, the overall pooled C-statistic of the machine learning-based algorithms was 0.76 [0.71–0.80], compared with an overall pooled C-statistic of 0.75 [0.73–0.77] in the traditional regression-based models. This information is presented in two forest plots (Supplementary Figures S1 and S2), the most popular way of graphically representing meta-analysis results^62^. The pooled effect size (C-statistic) and individual effect sizes (C-statistics) from each included study that predicted hypertension were graphically displayed in the forest plot.

In the past, several machine learning algorithms were developed for predicting hypertension^24–35^. Most of those algorithms used cross-sectional data and did not predict hypertension incidence. Some of the models used longitudinal data but did not incorporate time into their model. Only two models predicted the incidence of hypertension, considering survival data using machine learning algorithms^29,63^. Ye et al.^29^ used XGBoost, and Völzke et al.^63^ used the Bayesian network to build their model for predicting incident hypertension. However, neither study compared their model performance with conventional regression-based models. There have been only two studies^27,35^ where both conventional regression-based and machine learning-based models were developed simultaneously. Huang et al.^27^ and Farran et al.^35^ both created machine learning algorithms along with a conventional logistic regression model. Huang et al.^27^ used AUC to assess their models’ performance and found the artificial neural network’s AUC (0.90 ± 0.01) much higher than the logistic regression model’s AUC (0.73 ± 0.03). Farran et al.^35^ used classification accuracy to assess their models’ performance and found logistic regression had relatively similar accuracy (82.4) to other machine learning algorithms (82.4 ± 0.6 for support vector machines, 80.0 ± 0.8 for the k-Nearest neighbors, and 80.9 for multifactor dimensionality reduction). Nevertheless, none of the studies considered survival data in their modeling.

We employed feature selection methods before model building, and five different methods selected the top 20 features although feature space was not high-dimensional in our study, and penalized algorithms are already equipped to deal with high-dimensional data. However, having access to the entire set of features during model building has the disadvantage of sometimes including very irrelevant features in the final selected model because different machine learning techniques use different mechanisms for feature selection during their model building process. We intended to exclude very unimportant features from the model-building process. We noticed considerable variations in the top 20 features, as a result, we used a strategy in which higher-ranked features, which are common in all feature selection approaches, were allowed to be considered in the model-building process. By doing so, we ensured that the most irrelevant features were not examined by any of the feature selection procedures and that the most extreme irrelevant features were not included in the final model. We believe selecting common features made our model robust. Yet after employing feature selection methods, we discovered that different models assigned varying degrees of importance to various features. For example, feature CVD was given a high priority in penalized models but a low priority in gradient boosting (Fig. 2).

The relative importance of the features in predicting hypertension incidence revealed that waist-hip ratio, cardiovascular disease, diabetes, SBP, age, and BMI are the essential features. There are apparent discrepancies in a feature’s importance by different methods. DBP was identified as an important feature by RSF and GB. However, negligible importance was assigned to it in the penalized models. Perhaps this is due to its high collinearity with SBP, and penalized models tend to eliminate correlated features. Cardiovascular disease and diabetes were the two critical features identified in our study for predicting hypertension incidence, often avoided by most studies. This is because participants with cardiovascular disease and diabetes are often excluded from the model-building process in those studies.

Whether it is fair to compare multiple algorithms in the computational sciences and draw conclusions based on that comparison, and if so, under what situations and conditions this comparison should be undertaken and how it should be implemented, is the subject of some debate. Most commonly, studies are focused on the development of new methods and regularly contrast the new method with current methods, which may be heavily biased in favor of the new approach and should not be recognized as comparison studies since they are not neutral^64,65^. Neutral comparison studies that are devoted to the comparison itself do not seek to demonstrate the superiority of a certain method and may therefore be regarded as unbiased^64,65^. Such neutral comparative studies are crucial for the objective evaluation of existing methods, and their conduct is widely recommended^64,65^. However, they are conducted less frequently since many journals and journal editors view them as less attractive and less informative^64,65^. Although no precise criteria exist for how these comparative studies should be conducted, which competing approaches should be examined, or how they should be reported, Boulesteix et al.^65^ established three plausible requirements for a comparison study to meet in order to be considered neutral, as well as explaining general thoughts on the various components of a neutral comparison study. The requirements they establish are as follows: the primary objective of the study should be comparison itself; the authors should be reasonably neutral; and the assessment criteria, methodologies, and data sets selected should be rational. According to these three criteria, the execution of this comparison study was fair in the sense that the main objective of our study was comparison, and the authors were also sufficiently neutral. We also fared well in the third criterion, which comprises the selection of evaluation criteria, methods, and datasets. We used the C-index, a neutral and objective simple criterion, to evaluate our algorithms. Although the approaches were chosen subjectively, they were driven by objective factors such as the popularity of the models in practice and the findings accessible in the literature. In terms of data set selection, we attempted to select data (ATP) that is typical of the topic of our interest (the Canadian population). However, we used only one dataset for our comparative analysis, which may affect the findings' generalizability, and this is a potential weakness of this study.

This study’s unique strength is comparing machine learning algorithms with the conventional regression-based Cox model to predict hypertension incidence using survival data. To the best of our knowledge, this is the first time a comparison between machine learning algorithms and conventional regression models has been performed to predict hypertension incidence in survival data. Using large cohort data and considering many features is also a significant strength of this study. Notwithstanding the strengths, this study also has some limitations. Our study’s incidence rate of hypertension was relatively low compared to what is reported for the general Alberta population^66^. There can be several potential reasons for that. The characteristics of the study participants in ATP may be different from the general Alberta population. For example, female participation in ATP data was more than double the male participation (69% vs. 31%), and the hypertension incidence rate in Alberta was much lower in females than the males in study age groups^66^. A potential selection bias also may lead to a lower incidence rate of hypertension in our study. A selection bias is an error associated with recruiting study participants or factors affecting the study participation and usually occurs when selecting participants is not random^67^. The participants in ATP were mainly selected using the volunteer sampling method^68^. Those who decided to join the study (i.e., who self-select into the survey) may have a different characteristic (e.g., healthier) than the non-participants. Due to the longitudinal nature of the study, there can also be a loss of study participants during follow-up. Participants lost to follow-up (e.g., due to emigration out of the province) may be more likely to develop hypertension. Our study ascertained outcome hypertension from linked administrative health data (the hospital discharge abstract or physician claims data source) due to a lack of follow-up information in ATP. There is a possibility that the outcome ascertainment was incomplete. After cohort enrollment, people who did not have a healthcare encounter (e.g., did not visit a family physician/general practitioner or were not admitted to the hospital during the study period) were missed. Also, people may have seen their family doctor for a reason not primarily related to BP (e.g., they went to the family doctor for an upper respiratory tract infection) and consequently their BP may not recorded. All these can potentially lead to a lower hypertension incidence. We only compared C-index to evaluate the models’ predictive performance. We basically intended to assess the predictive performance of various algorithms using a generally recognized standard metric. Given this, the C-index was the obvious choice as C-index is the most commonly used predictive measure. We would prefer to compare the predictive performances of all algorithms using a standard calibration metric as well (e.g., Brier score). However, a common calibration metric under all the settings studied in this study was not available, either in a software program or simply not developed. We also did not use feature scaling approaches for continuous features, such as standardization or normalization, which may have an impact on the predictive performance of some of the algorithms. We could not evaluate our models’ performance in an external cohort, which is essential for any prediction model’s generalizability^7^. The current study had a limited focus. We only used a subset of machine learning algorithms and hence cannot comment on the performance of approaches not tested here, such as neural networks and support vector machines. Our findings about the relative performance of various prediction methods should be limited to this patient cohort and this specific prediction (i.e., hypertension ). Readers should not draw the conclusion that traditional statistical modeling and machine learning algorithms perform similarly in all scenarios and for all conditions or outcomes.

In conclusion, we developed several machine learning algorithms for predicting hypertension incidence using survival data. We compared machine learning algorithms’ performance with conventional Cox PH regression models, and a negligible difference in predictive performance was observed. Based on this study’s findings, conventional regression-based models are comparable to machine learning algorithms to provide good predictive accuracy in a moderate dataset with a reasonable number of features.

## Supplementary Information

Supplementary Information.

## Data Availability

The data that support the findings of this study are available from Alberta’s Tomorrow Project (ATP) but restrictions apply to the availability of these data, which were used under license for the current study, and so are not publicly available. Data are however available from the authors upon reasonable request and with permission of Alberta’s Tomorrow Project (ATP).

## References

[1] World Health Organization (2014). Global Status Report on noncommunicable diseases 2014—Quot; Attaining the nine global noncommunicable diseases targets; a shared responsibility & quot.

[2] Zhou B, Carrillo-Larco RM, Danaei G (2021). Worldwide trends in hypertension prevalence and progress in treatment and control from 1990 to 2019: A pooled analysis of 1201 population-representative studies with 104 million participants. Lancet.

[3] Zhou B, Perel P, Mensah GA, Ezzati M (2021). Global epidemiology, health burden and effective interventions for elevated blood pressure and hypertension. Nat. Rev. Cardiol..

[4] The effects of hypertension on the body. Accessed January 2, 2021. https://www.healthline.com/health/high-blood-pressure-hypertension/effect-on-body

[5] Ahmed I, Debray TP, Moons KG, Riley RD (2014). Developing and validating risk prediction models in an individual participant data meta-analysis. BMC Med. Res. Methodol..

[6] Chowdhury MZI, Turin TC (2020). Precision health through prediction modelling: Factors to consider before implementing a prediction model in clinical practice. J. Prim. Health Care.

[7] Chowdhury MZI, Turin TC (2021). Validating prediction models for use in clinical practice: Concept, steps, and procedures focusing on hypertension risk prediction. Hypertens. J..

[8] Chowdhury MZI, Naeem I, Quan H (2020). Summarising and synthesising regression coefficients through systematic review and meta-analysis for improving hypertension prediction using metamodelling: Protocol. BMJ Open.

[9] Chowdhury MZI, Naeem I, Quan H (2022). Prediction of hypertension using traditional regression and machine learning models: A systematic review and meta-analysis. PLoS One.

[10] Chowdhury MZI, Turin TC (2020). Variable selection strategies and its importance in clinical prediction modelling. Fam. Med. Community Health.

[11] Chowdhury MZI, Leung AA, Sikdar KC, O’Beirne M, Quan H, Turin TC (2022). Development and validation of a hypertension risk prediction model and construction of a risk score in a Canadian population. Sci. Rep..

[12] Steyerberg EW, van der Ploeg T, Van Calster B (2014). Risk prediction with machine learning and regression methods. Biomet. J..

[13] Wang P, Li Y, Reddy CK (2017). Machine learning for survival analysis: A survey. arXiv..

[14] Framingham T, Study H (2017). Article annals of internal medicine a risk score for predicting near-term incidence of hypertension. Ann. Intern. Med..

[15] Kanegae H, Oikawa T, Suzuki K, Okawara Y, Kario K (2018). Developing and validating a new precise risk-prediction model for new-onset hypertension: The Jichi Genki hypertension prediction model (JG model). J Clin. Hypertens..

[16] Chen Y, Wang C, Liu Y (2016). Incident hypertension and its prediction model in a prospective northern urban Han Chinese cohort study. J. Hum. Hypertens..

[17] Lim NK, Son KH, Lee KS, Park HY, Cho MC (2013). Predicting the risk of incident hypertension in a Korean middle-aged population: Korean genome and epidemiology study. J. Clin. Hypertens..

[18] Pearson TA, LaCroix AZ, Mead LA, Liang KY (1990). The prediction of midlife coronary heart disease and hypertension in young adults: The Johns Hopkins multiple risk equations. Am. J. Prev. Med..

[19] Paynter NP, Cook NR, Everett BM, Sesso HD, Buring JE, Ridker PM (2009). Prediction of incident hypertension risk in women with currently normal blood pressure. Am. J. Med..

[20] Zhang W, Wang L, Chen Y, Tang F, Xue F, Zhang C (2015). Identification of hypertension predictors and application to hypertension prediction in an urban Han Chinese population: A longitudinal study, 2005–2010. Prev. Chronic Dis..

[21] Wang B, Liu Y, Sun X (2020). Prediction model and assessment of probability of incident hypertension: The rural Chinese Cohort study. J. Hum. Hypertens..

[22] Otsuka T, Kachi Y, Takada H (2015). Development of a risk prediction model for incident hypertension in a working-age Japanese male population. Hypertens. Res..

[23] Kadomatsu Y, Tsukamoto M, Sasakabe T (2019). A risk score predicting new incidence of hypertension in Japan. J. Hum. Hypertens..

[24] Sakr S, Elshawi R, Ahmed A (2018). Using machine learning on cardiorespiratory fitness data for predicting hypertension: The Henry Ford exercise testing (FIT) project. PLoS ONE.

[25] Kwong EWY, Wu H, Pang GKH (2018). A prediction model of blood pressure for telemedicine. Health Inform. J..

[26] Polak S, Mendyk A (2008). Artificial neural networks based Internet hypertension prediction tool development and validation. Appl. Soft. Comput. J..

[27] Huang S, Xu Y, Yue L (2010). Evaluating the risk of hypertension using an artificial neural network method in rural residents over the age of 35 years in a Chinese area. Hypertens. Res..

[28] Falk CT (2003). Risk factors for coronary artery disease and the use of neural networks to predict the presence or absence of high blood pressure. BMC Genet..

[29] Ye C, Fu T, Hao S (2018). Prediction of incident hypertension within the next year: Prospective study using statewide electronic health records and machine learning. J. Med. Internet Res..

[30] Priyadarshini R, Barik RK, Dubey H (2018). DeepFog: Fog computing-based deep neural architecture for prediction of stress types, diabetes and hypertension attacks. Computation.

[31] Wu, T. H., Kwong, E. W. Y, Pang, G. K. H. (2015) Bio-medical application on predicting systolic blood pressure using neural networks. *Proc.—2015 IEEE 1st International Conference on Big Data Computing Service and Application*. pp. 456–461 10.1109/BigDataService.2015.54

[32] Wu, T. H., Pang, G. K. H., Kwong, E. W. Y. (2014) Predicting systolic blood pressure using machine learning. *2014 7th International Conf. Informatiom and Automation Sustainability ICIAfS* pp. 1–6 10.1109/ICIAFS.2014.7069529

[33] Tayefi M, Esmaeili H, Saberi Karimian M (2017). The application of a decision tree to establish the parameters associated with hypertension. Comput. Methods Programs Biomed..

[34] Zhang B, Wei Z, Ren J, Cheng Y, Zheng Z (2018). An empirical study on predicting blood pressure using classification and regression trees. IEEE Access..

[35] Farran B, Channanath AM, Behbehani K, Thanaraj TA (2013). Predictive models to assess risk of type 2 diabetes, hypertension and comorbidity: Machine-learning algorithms and validation using national health data from Kuwait-a cohort study. BMJ Open.

[36] Kruppa J, Liu Y, Biau G (2014). Probability estimation with machine learning methods for dichotomous and multicategory outcome: Theory. Biom. J..

[37] Van Hoorde K, Van Huffel S, Timmerman D, Bourne T, Van Calster B (2015). A spline-based tool to assess and visualize the calibration of multiclass risk predictions. J. Biomed. Inform..

[38] Desai RJ, Wang SV, Vaduganathan M, Evers T, Schneeweiss S (2020). Comparison of machine learning methods with traditional models for use of administrative claims with electronic medical records to predict heart failure outcomes. JAMA Netw. Open..

[39] Austin PC, Tu JV, Ho JE, Levy D, Lee DS (2013). Using methods from the data-mining and machine-learning literature for disease classification and prediction: A case study examining classification of heart failure subtypes. J. Clin. Epidemiol..

[40] Tollenaar N, van der Heijden PGM (2013). Which method predicts recidivism best?: A comparison of statistical, machine learning and data mining predictive models. J. R. Stat. Soc. Ser. A Stat. Soc..

[41] Song X, Mitnitski A, Cox J, Rockwood K (2004). Comparison of machine learning techniques with classical statistical models in predicting health outcomes. Stud. Health Technol. Inform..

[42] Frizzell JD, Liang L, Schulte PJ (2017). Prediction of 30-day all-cause readmissions in patients hospitalized for heart failure: Comparison of machine learning and other statistical approaches. JAMA Cardiol..

[43] Van Buuren S, Boshuizen HC, Knook DL (1999). Multiple imputation of missing blood pressure covariates in survival analysis. Stat. Med..

[44] van Buuren S, Oudshoorn CGM (2007). MICE: Multivariate imputation by chained equations inR. Package Ver..

[45] Chowdhury MZI (2021). Develop a Comprehensive Hypertension Prediction Model and Risk Score in Population-based Data Applying Conventional Statistical and Machine Learning Approaches.

[46] Quan H, Khan N, Hemmelgarn BR (2009). Validation of a case definition to define hypertension using administrative data. Hypertension.

[47] Chandrashekar G, Sahin F (2014). A survey on feature selection methods. Comput. Electr. Eng..

[48] Lang M, Kotthaus H, Marwedel P, Weihs C, Rahnenführer J, Bischl B (2015). Automatic model selection for high-dimensional survival analysis. J. Stat. Comput. Simul..

[49] Tsamardinos I, Brown LE, Aliferis CF (2006). The max-min hill-climbing Bayesian network structure learning algorithm. Mach. Learn..

[50] Breiman, L. (2001) Random forests. *Mach. Learn.*10.1023/A:1010933404324

[51] Max, A., Wing, J., Weston, S. et al. (2020) Package ‘caret’ R. **223**.

[52] Zihni E, Madai VI, Livne M (2020). Opening the black box of artificial intelligence for clinical decision support: A study predicting stroke outcome. PLoS One..

[53] Harrell FE, Califf RM, Pryor DB, Lee KL, Rosati RA (1982). Evaluating the Yield of Medical Tests. JAMA J. Am. Med. Assoc..

[54] Tsagris M, Papadovasilakis Z, Lakiotaki K, Tsamardinos I (2018). Efficient feature selection on gene expression data: Which algorithm to use?. bioRxiv..

[55] Jerome, A., Hastie, T., Tibshirani, R., Tay, K., Simon, N. (2020) Package ‘glmnet’ R topics documented : Published online.

[56] Learning, T. M., Interface, D., Bsd, L., Url, L., Paramhelpers, D. (2020) Suggests XML. Package ‘Mlr’.

[57] Lumley, T. S-. R., Elizabeth, A., Cynthia, C., Therneau, M. T. M. (2020) Package ‘survival’. Published online.

[58] Greenwell, B., Boehmke, B., Cunningham, J. (2019) Package “gbm”—Generalized boosted regression models. CRAN Repos. Published online 2019:39. https://cran.r-project.org/web/packages/gbm/gbm.pdf, https://github.com/gbm-developers/gbm

[59] Boosting, T. M., Matrix, I. (2020) *Package ‘Mboost’*. 10.1007/s00180-012-0382-5

[60] van Buuren S, Groothuis-Oudshoorn K (2011). Mice: Multivariate imputation by chained equations in R. J. Stat. Softw. Pub. Online.

[61] Hosmer DW, Lemeshow S, Sturdivant RX (2013). Applied Logistic Regression.

[62] Chowdhury, M. Z. I, Turin, T. (2019) Synthesizing quantitative and qualitative studies in systematic reviews: The basics of meta-analysis and meta-synthesis. J. Natl. Hear Found Bangladesh. https://www.nhf.org.bd/Journal/Web_Upload/JNHFB_2019/2019_JNHFB_Vol 8 Issue 2/4 Synthesizing Quantitative and Qualitative Studies.pdf

[63] Völzke H, Fung G, Ittermann T (2013). A new, accurate predictive model for incident hypertension. J. Hypertens. Pub. Online.

[64] Boulesteix AL, Binder H, Abrahamowicz M, Sauerbrei W (2018). On the necessity and design of studies comparing statistical methods. Biom. J..

[65] Boulesteix AL, Lauer S, Eugster MJA (2013). A plea for neutral comparison studies in computational sciences. PLoS One.

[66] Interactive health data application—Display results. Accessed March 29, 2021. http://www.ahw.gov.ab.ca/IHDA_Retrieval/selectSubCategoryParameters.do

[67] Tripepi G, Jager KJ, Dekker FW, Zoccali C (2010). Selection bias and information bias in clinical research. Nephron Clin. Pract..

[68] Ye M, Robson PJ, Eurich DT, Vena JE, Xu JY, Johnson JA (2017). Cohort profile: Alberta’s tomorrow project. Int. J. Epidemiol..

