# Supplementary material for: A comparison of machine learning algorithms and traditional regression-based statistical modeling for predicting hypertension incidence in a Canadian population
Source: Sci Rep. 2023 Jan 2;13:13. doi: 10.1038/s41598-022-27264-x (PMC9807553; doi:10.1038/s41598-022-27264-x)
Supplement: Supplementary file 1 — Supplementary Information. [file 41598_2022_27264_MOESM1_ESM.docx]

**SUPPLEMENTARY MATERIALS**

**SUPPLEMENTARY FIGURE LEGENDS**

**Figure S1.** Forest plot of traditional regression-based models with 95% prediction interval

**Figure S2.** Forest plot of machine learning-based models with 95% prediction interval

**Figure S1.**

**
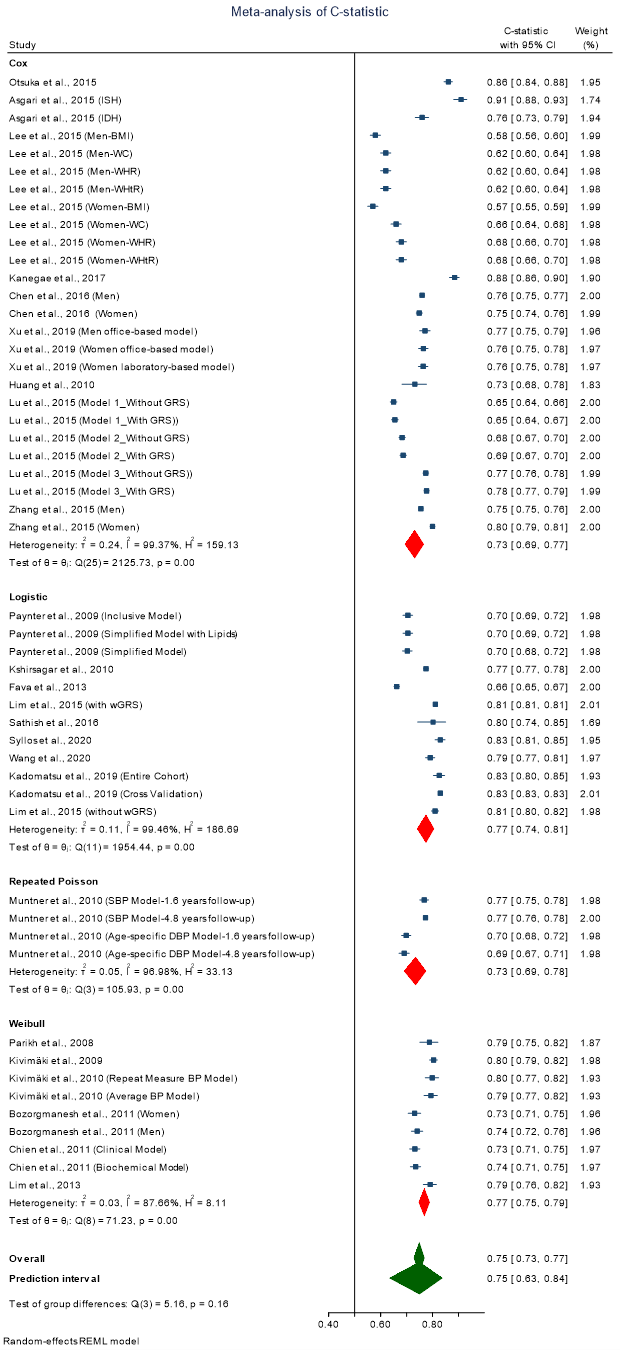
**

**Figure S2.**


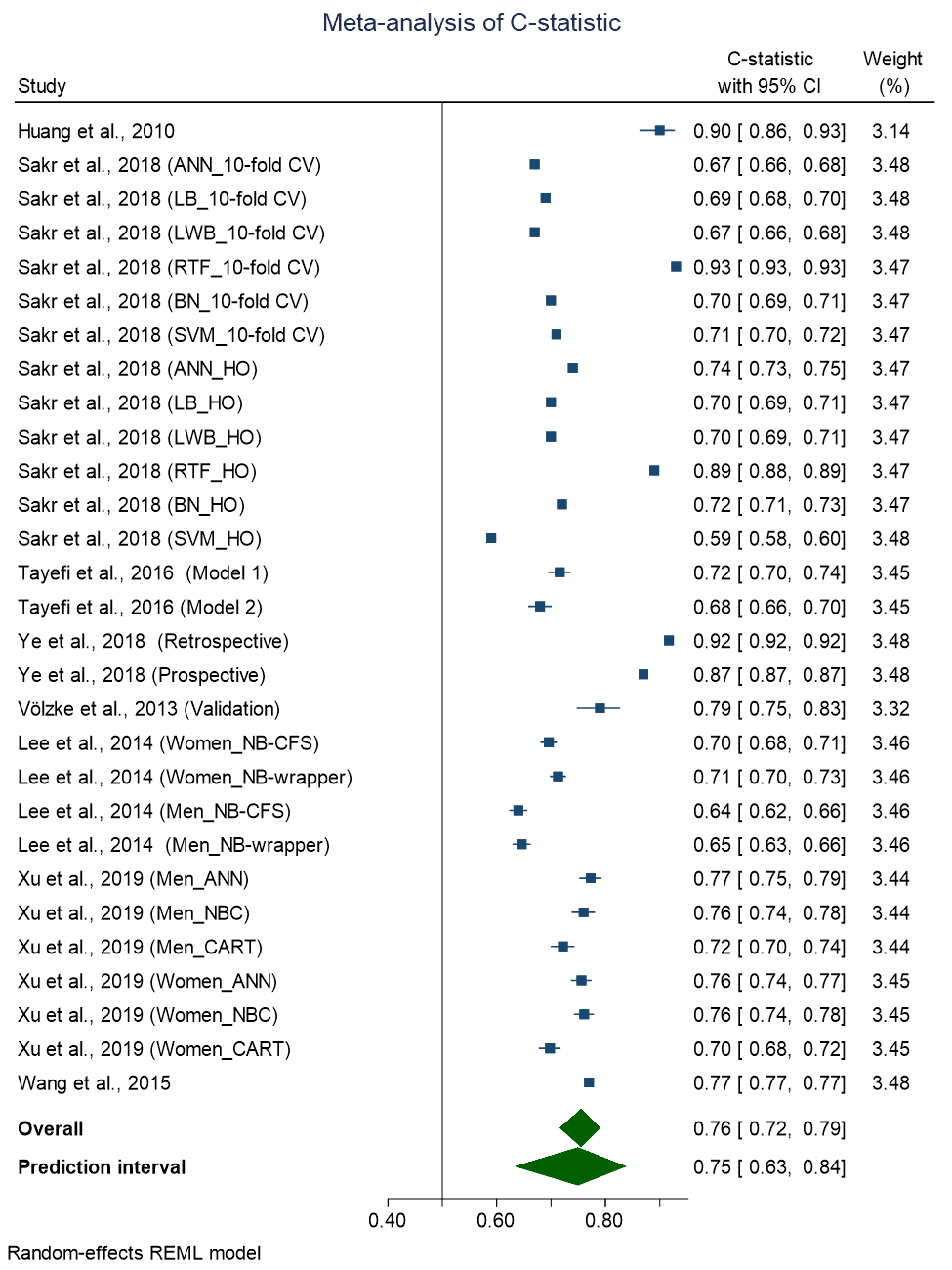


**Table S1.** Missing information about different variables

| **Variables** | **Missing** | **Total** | **Percent Missing** |
| --- | --- | --- | --- |
| Total Physical Activity Time | 520 | 18,322 | 2.84 |
| Total Sitting Time | 1,421 | 18,322 | 7.76 |
| Depression | 16 | 18,322 | 0.09 |
| Diabetes | 8 | 18,322 | 0.04 |
| Waist Hip Ratio | 4,686 | 18,322 | 25.58 |
| Sex | 0 | 18,322 | 0.00 |
| Age | 0 | 18,322 | 0.00 |
| Residence | 0 | 18,322 | 0.00 |
| Family History of Hypertension | 0 | 18,322 | 0.00 |
| Diastolic Blood Pressure | 4,283 | 18,322 | 23.38 |
| Systolic Blood Pressure | 4,283 | 18,322 | 23.38 |
| Ethnicity | 23 | 18,322 | 0.13 |
| Cardiovascular Disease | 0 | 18,322 | 0.00 |
| Highest Education Level Completed | 11 | 18,322 | 0.06 |
| Working Status | 0 | 18,322 | 0.00 |
| Vegetable and Fruit Consumption | 266 | 18,322 | 1.45 |
| Physical Activity | 1,846 | 18,322 | 10.08 |
| Total Household Income | 1,402 | 18,322 | 7.65 |
| Alcohol Consumption | 846 | 18,322 | 4.62 |
| Total Sleep Time | 239 | 18,322 | 1.30 |
| Smoking Status | 45 | 18,322 | 0.25 |
| Job Schedule | 4,303 | 18,322 | 23.49 |
| Marital Status | 7 | 18,322 | 0.04 |
| Body Mass Index | 4,260 | 18,322 | 23.25 |
| BMI Waist Ratio | 4,718 | 18,322 | 25.75 |
| Ever Smoked | 41 | 18,322 | 0.22 |
| Body Fat Percentage | 4,471 | 18,322 | 24.40 |
| Hip Circumference | 4,564 | 18,322 | 24.91 |
| Waist Circumference | 4,769 | 18,322 | 26.03 |

**Table S2.** Baseline characteristics of study participants and comparison of the training and test data

| **Socio-demographic characteristics of groups** | | | | | |
| --- | --- | --- | --- | --- | --- |
| **Variable** | **Categories** | **All participants**  **(18,322)** | **Derivation sample**  **(n =** **12,233)** | **Validation sample**  **(n =** **6,089)** | **P-value** |
| Age, years, mean (SE) |  | 50.99 (9.20) | 50.94 (9.19) | 51.07 (9.24) | 0.377 |
| Sex, n (%) | Male (reference) | 5,763 (31.45) | 3844 (31.42) | 1919 (31.52) | 0.899 |
|  | Female | 12,559 (68.55) | 8389 (68.58) | 4170 (68.48) |  |
| Body Mass Index, kg/m2, mean (SE) |  | 26.45 (4.90) | 26.48 (4.94) | 26.39 (4.81) | 0.582 |
| Waist-Hip Ratio, mean (SE) |  | 0.91 (0.07) | 0.91 (0.07) | 0.91 (0.07) | 0.882 |
| Diastolic Blood Pressure, mean (SE) |  | 72.95 (9.35) | 72.93 (9.35) | 72.97 (9.34) | 0.787 |
| Systolic Blood Pressure, mean (SE) |  | 119.81 (13.73) | 119.75 (13.73) | 119.92 (13.71) | 0.446 |
| Marital Status, n (%) | Married and/or living with a partner (reference) | 14,458 (78.91) | 9659 (78.96) | 4799 (78.81) | 0.226 |
|  | Single, never married | 1180 (6.44) | 763 (6.24) | 417 (6.85) |  |
|  | Other (divorced, widowed, separated) | 2684 (14.65) | 1811 (14.80) | 873 (14.34) |  |
| Residence, n (%) | Urban (reference) | 15,272 (83.35) | 10,180 (83.22) | 5092 (83.63) | 0.484 |
|  | Rural | 3050 (16.65) | 2053 (16.78) | 997 (16.37) |  |
| Total Household Income, n (%) | < $49,999 (reference) | 2855 (15.58) | 1904 (15.56) | 951 (15.62) | 0.416 |
|  | $50,000 - $99,999 | 5889 (32.14) | 3902 (31.90) | 1987 (32.63) |  |
|  | $100,000 - $199,999 | 7149 (39.02) | 4823 (39.43) | 2326 (38.20) |  |
|  | ≥ $200,000 | 2429 (13.26) | 1604 (13.11) | 825 (13.55) |  |
| Highest Education Level Completed, n (%) | High school or below (none, elementary school, high school, trade, technical or vocational school, apprenticeship training or technical CEGEP) (reference) | 6161 (33.63) | 4073 (33.30) | 2088 (34.29) | 0.310 |
|  | Diploma but below bachelor’s degree (diploma from a community college, pre-university CEGEP or non-university certificate, university certificate below bachelor’s level) | 4928 (26.90 | 3288 (26.88) | 1640 (26.93) |  |
|  | Bachelor’s degree or above (bachelor's degree, graduate degree (MSc, MBA, MD, PhD, etc.)) | 7233 (39.48) | 4872 (39.83) | 2361 (38.77) |  |
| Ethnicity, n (%) | Aboriginal | 68 (0.37) | 49 (0.40) | 19 (0.31) | 0.316 |
|  | Asian (South Asian, East Asian, Southeast Asian, Filipino, West Asian, Arab) | 827 (4.51) | 545 (4.46) | 282 (4.63) |  |
|  | White (reference) | 16,895 (92.21) | 11,274 (92.16) | 5621 (92.31) |  |
|  | Latin American Hispanic | 162 (0.88) | 121 (0.99) | 41 (0.67) |  |
|  | Black | 97 (0.53) | 63 (0.52) | 34 (0.56) |  |
|  | Other (Jewish and others) | 273 (1.49) | 181 (1.48) | 92 (1.51) |  |
| Diabetes, n (%) |  | 735 (4.01) | 502 (4.10) | 233 (3.83) | 0.368 |
| Cardiovascular Disease, n (%) |  | 377 (2.06) | 257 (2.10) | 120 (1.97) | 0.559 |
| Depression, n (%) |  | 2013 (10.99) | 1366 (11.17) | 647 (10.63) | 0.270 |
| Family History of Hypertension, n (%) |  | 10,946 (59.74) | 7266 (59.40) | 3680 (60.44) | 0.176 |
| Smoking Status, n (%) | Never (reference) | 10,116 (55.21) | 6739 (55.09) | 3377 (55.46) | 0.763 |
|  | Former | 6763 (36.91) | 4537 (37.09) | 2226 (36.56) |  |
|  | Current | 1443 (7.88) | 957 (7.82) | 486 (7.98) |  |
| Alcohol Consumption, n (%) | Never (reference) | 1293 (7.06) | 869 (7.10) | 424 (6.96) | 0.855 |
|  | ≤ 1 time a week | 9644 (52.64) | 6415 (52.44) | 3229 (53.03) |  |
|  | 2 to 3 times a week | 3807 (20.78) | 2535 (20.72) | 1272 (20.89) |  |
|  | 4 to 5 times a week | 1993 (10.88) | 1340 (10.95) | 653 (10.72) |  |
|  | ≥ 6 times a week | 1585 (8.65) | 1074 (8.78) | 511 (8.39) |  |
| Working Status, n (%) | Full time (reference) | 10,281 (56.11) | 6836 (55.88) | 3445 (56.58) | 0.065 |
|  | Part time | 3719 (20.30) | 2543 (20.79) | 1176 (19.31) |  |
|  | Other (looking after home, disable/sick, student, unpaid/voluntary) | 3974 (21.69) | 2614 (21.37) | 1360 (22.34) |  |
|  | Unemployed | 348 (1.90) | 240 (1.96) | 108 (1.77) |  |
| Total Sleep Time, n (%) | ≤ 5 hours (short sleep duration) | 1191 (6.50) | 804 (6.57) | 387 6.36 | 0.257 |
|  | 6 hours | 3739 (20.41) | 2441 (19.95) | 1298 (21.32) |  |
|  | 7 hours (reference) | 7042 (38.43) | 4747 (38.80) | 2295 (37.69) |  |
|  | 8 hours | 5111 (27.90) | 3414 (27.91) | 1697 (27.87) |  |
|  | ≥ 9 hours (long sleep duration) | 1239 (6.76) | 827 (6.76) | 412 (6.77) |  |
| Total Physical Activity Time, mean (SE) |  | 3158.53 (2869.02) | 3157.97 (2853.36) | 3159.66 (2900.45) | 0.970 |
| Total Sitting Time, mean (SE) |  | 2487.77 (1174.02) | 2495.39 (1176.80) | 2472.48 (1168.35) | 0.214 |
| Physical Activity, n (%) | Low (first quartile of physical activity time and fourth quartile of sitting time) (reference) | 1691 (9.23) | 1157 (9.46) | 534 (8.77) | 0.280 |
|  | Moderate (second and third quartile of physical activity time and sitting time) | 14,479 (79.03) | 9653 (78.91) | 4826 (79.26) |  |
|  | High (fourth quartile of physical activity and first quartile of sitting time) | 2152 (11.75) | 1423 (11.63) | 729 (11.97) |  |
| Vegetable and Fruit Consumption, n (%) | Low consumption (less than 5 servings of vegetables and fruit) (reference) | 15,273 (83.36) | 10,182 (83.23) | 5091 (83.61) | 0.620 |
|  | Moderate consumption (less than 5 servings of vegetables but more than 5 servings of fruit OR more than 5 servings of vegetables but less than 5 servings of fruits) | 2529 (13.80) | 1694 (13.85) | 835 (13.71) |  |
|  | High consumption (5 or more servings of vegetables and fruit) | 520 (2.84) | 357 (2.92) | 163 (2.68) |  |
| Job Schedule, n (%) | Regular daytime shift (reference) | 11,920 (65.06) | 7985 (65.27) | 3935 (64.62) | 0.385 |
|  | Other (evening shift, night shift, rotating shift, split shift, irregular shift, or on-call) | 6402 (34.94) | 4248 (34.73) | 2154 (35.38) |  |

**APPENDIX 1.**

**Alberta’s Tomorrow Project (ATP) cohort data**

Alberta’s Tomorrow Project (ATP) cohort data is a province-wide prospective cohort study and consists of Alberta’s residents, aged 35-69 years, without any history of cancer other than non-melanoma skin cancer^1^. ATP is a part of a pan-Canadian initiative to investigate the causes and prevention of cancer and chronic diseases. Launched in 2000, ATP is Alberta’s largest longitudinal population health cohort from the general population. It contains baseline and longitudinal information on socio-demographic characteristics, personal and family history of the disease, medication use, lifestyle and health behavior, environmental exposures, and physical measures. ATP joined the Canadian Partnership for Tomorrow Project (CPTP) in 2008^2^. ATP had three baseline questionnaires: Canadian Diet History Questionnaire-I (CDHQ-I), Health and Lifestyle Questionnaire (HLQ), and the Past-Year Total Physical Activity Questionnaire (PYTPAQ), and two follow-up questionnaires: Survey 2004 and Survey 2008, during the period 2001-2008. When ATP merged with CPTP, participants were asked to complete two versions of questionnaires: The Updated Health and Lifestyle Questionnaire (UHLQ), along with the Physical Activity and Nutrition Survey (PANS) or the CORE questionnaire^3^. Participants completed either UHLQ/PANS or CORE because both questionnaires contained very similar information. UHLQ/PANS or CORE questionnaires were more elaborate and captured more information about the participants than the other questionnaires.

The recruitment of participants in ATP was done in two phases^4^. In Phase I (2000-08), participants were recruited using a two-stage telephone-based random digit dialing method^1^. Eight waves of telephone-based random digit dialing (RDD) using Alberta’s regional health authority boundaries as the sampling frame was used to recruit participants^1^. Participants were identified using a 2-stage method. In the first stage, a household was identified, and in the second stage, one or two eligible adults within the identified household were selected for participation^1^. Participants selected from the same household a second time were excluded to avoid repetition^4^. In Phase I, 29,878 participants were recruited with a response rate of 49%^4^.

In Phase II (2009-15), when ATP joined with the Canadian Partnership for Tomorrow Project (CPTP)–an alliance of five cohorts across Canada (British Columbia, Alberta, Ontario, Quebec, and Atlantic Canada), ATP-CPTP recruitment began using a volunteer sampling method^4^. Existing ATP participants (Phase I participants) were invited to join CPTP and requested to visit study centers for physical measurements and blood and urine contributions^4^. Fifteen thousand one hundred sixty-two participants from Phase I (approximately 50%) agreed to join CPTP, of which about 60% visited Study Centres^4^. Due to ATP’s pledge to enroll roughly 40,000 participants to CPTP from Alberta, more participants were recruited. Nevertheless, the process for selecting potential participants in CPTP varies between jurisdictions. It includes a random selection from population-based data, purchase of mailing lists for specific geographic areas, RDD, and word of mouth^5^. Telephone-based RDD was initially used to recruit new ATP-CPTP participants in 2009 but was soon replaced by volunteer sampling due to the low response rate and increasing cost^4^. Further communication and advocating strategies were employed to promote volunteer recruitment, such as marketing, advertising, media coverage, information booths at community events, corporate presentations, Ambassador Program, and articles^4^. In Phase II, 22,932 participants were recruited through volunteer sampling.

An invitation package was sent to the eligible participants (in both phases), including a cover letter, a study information booklet, an explicit consent to participate in the ATP and allow data linkage, and a self-administered ATP questionnaire^4^. Those who completed the ATP questionnaire and agreed to data linkage were considered ATP participants. By March 2015, 52,810 Alberta residents had signed up for the ATP and decided to have their data linked to healthcare databases, with 38,094 of them agreeing to participate in the CPTP as well^4^. Of the total 52,810 ATP participants, 29,878 completed HLQ, 25,955 completed CDHQ, 25,889 completed PYTPAQ, 8,540 completed Survey 2004, 20,107 completed Survey 2008, 12,395 completed UHLQ, 12,402 completed PNAS and 25,677 completed the CORE questionnaire^4^.

ATP was built to represent Alberta’s general population with no history of cancer other than non-melanoma skin cancer. To see how different ATP participants are from the rest of the Alberta population and compare their characteristics, a study was conducted where the ATP cohort was compared with the Alberta-specific subsets of Canadian Community Health Survey (CCHS) participants in the same age group (35-69 years). ATP participants were older, had more women, were more likely to be obese and less likely to smoke, ate more fruits and vegetables, and were more physically active than CCHS participants^4^. Corresponding to the two ATP recruitment phases, two different cycles of CCHS were used to make the comparison fair.

**REFERENCES**

1. Robson PJ, Solbak NM, Haig TR, et al. Design, methods and demographics from phase I of Alberta’s Tomorrow Project cohort: a prospective cohort profile. *C Open*. 2016;4(3):E515-E527. doi:10.9778/cmajo.20160005

2. Summary Data Tables | Alberta’s Tomorrow Project. Accessed December 15, 2020. http://myatp.ca/for-researchers/summary-data-tables

3. Survey Questions Asked - Alberta’s Tomorrow Project. Accessed January 4, 2021. https://myatpresearch.ca/survey-questions/

4. Ye M, Robson PJ, Eurich DT, Vena JE, Xu JY, Johnson JA. Cohort profile: Alberta’s Tomorrow Project. *Int J Epidemiol*. 2017;46(4):1097-1098l. doi:10.1093/ije/dyw256

5. Borugian MJ, Robson P, Fortier I, et al. The Canadian Partnership for Tomorrow Project: Building a pan-Canadian research platform for disease prevention. *Cmaj*. 2010;182(11):1197-1201. doi:10.1503/cmaj.091540

**APPENDIX 2.**

**Feature’s Description**

The continuous features (e.g., age, BMI, SBP, etc.) remain continuous. The rest of the features were categorical. The sex of the participants was either male or female. The residence was either urban or rural. Marital status was categorized into three groups: married and/or living with a partner, single who never married, and others (divorced, widowed, separated). Total household income was categorized into four groups: < $49,999, $50,000-$99,999, $100,000-$199,999, and ≥ $200,000. The highest education level completed was categorized into three groups: high school or below (none, elementary school, high school, trade, technical or vocational school, apprenticeship training or technical CEGEP), diploma but below bachelor’s degree (diploma from a community college, pre-university CEGEP or non-university certificate, university certificate below bachelor’s level), and bachelor’s degree or above (bachelor’s degree, graduate degree [MSc, MBA, MD, PhD, etc.]). Ethnicity was categorized into six groups: Aboriginal, Asian (South Asian, East Asian, Southeast Asian, Filipino, West Asian, Arab), White, Latin American Hispanic, Black, and other (Jewish and others). Diabetes was categorized as “yes” or “no” based on the response to the question, “Has a doctor ever told you that you had diabetes?”. Cardiovascular disease was categorized as “yes” if any stroke, myocardial infarction, angina, arrhythmia, coronary heart disease, coronary artery disease, heart disease, and heart failure was present and as ‘no” if absent. Depression was categorized as “yes” or “no” based on the response to the question “Has a doctor ever told you that you had depression?”. Family history of hypertension was categorized as “yes” if any first-degree relative was diagnosed with hypertension, otherwise “no”. Smoking status was categorized as: never, former, and current. Alcohol consumption was categorized into five groups: never, ≤ 1 time a week, 2 to 3 times a week, 4 to 5 times a week, and ≥ 6 times a week. Working status was categorized into four groups: full-time, part-time, other (looking after a home, disable/sick, student, unpaid/voluntary), and unemployed. Total sleep time was categorized into four groups: ≤ 5 hours (short sleep duration), 6 to 7 hours, 8 hours, and ≥ 9 hours (long sleep duration). Physical activity was categorized as: low (first quartile of physical activity time and fourth quartile of sitting time), moderate (second and third quartile of physical activity time and sitting time), and high (fourth quartile of physical activity and first quartile of sitting time). Vegetable and fruit consumption was categorized as low (less than 5 servings of vegetable and fruit), moderate (less than 5 servings of vegetable but more than 5 servings of fruit OR more than 5 servings of vegetable but less than 5 servings of fruits), and high (5 or more servings of vegetable and fruit). Job schedule was categorized as regular daytime shift and other (evening shift, night shift, rotating shift, split shift, irregular shift, or on-call).

**APPENDIX 3.**

**Feature Selection Techniques**

Modern-day datasets are rich in information, with data collected on many features, making the data high dimensional. Such high-dimensional datasets create computational difficulty and complicate the interpretability of a prediction model. Feature selection is a process where a subset of relevant features from a large amount of data is selected to filter the dataset down to the smallest possible subset of accurate features. It is imperative to identify the relevant features from a dataset and remove less significant features that have a minimal contribution to the outcome to achieve better prediction model accuracy. Feature selection is one of the core concepts in machine learning that massively impacts a model’s performance. Feature selection offers enhanced model performance by mitigating the risk of overfitting, improved computational speed and time, decreased computational requirements, and easier model interpretability.

Feature selection methods can be classified into three categories: filter, wrapper, and embedded methods^1^. Filter methods use feature ranking techniques as the main criteria for feature selection^1^. An appropriate ranking criterion is applied to score the features, and features below a specified threshold are eliminated. Filter methods serve as a preprocess to rank the features in which the highest-ranked features are selected. Wrapper methods use the performance of the model as the feature selection criterion^1^. The model is wrapped in a search algorithm to find a subset of the features that give the highest model performance. Embedded methods integrate the selection of features as part of the model building process^1^.

There are different ways to assign numerical scores within filter methods so that features can be ordered based on their relevance. Two popular variants of filter methods in the survival analysis setting are a univariate Cox p-value and C-index^2^. A univariate Cox model is separately applied for each feature, and p-values are obtained^3^. These p-values are used as importance scores. The C-index calculation is performed for each feature without fitting a survival model. The resulting C-index is used as a score for that feature^2^. Features are ordered according to their C-index, and a higher C-index indicates more importance. Two popular embedded methods of feature selection are RSF and Lasso. Both are machine learning approaches for building prediction models and performing feature selection. Variable importance in RSF is calculated using a prediction error approach involving noising up the feature by randomly permuting its value^4^. A feature’s variable importance is the difference between a prediction error when a feature is noised and a prediction error in the original feature^4^. The Lasso method shrinks the regression model’s coefficients as part of penalization, and the features left after the shrinkage process are selected for model building. In Lasso, prediction/fitting errors are minimized using the objective functions, and the features with near-zero regression coefficients are eliminated^5^. A constraint-based method for feature selection is the statistically equivalent signature (SES)^6^, which tries to identify multiple subsets of predictive features whose performance is statistically equivalent^7^. The signature here implies minimal sets of features with maximum predictive power. The primary purpose of running the SES algorithm is to select variables as important according to the increasing p-value.

**REFERENCES**

1. Chandrashekar G, Sahin F. A survey on feature selection methods. *Comput Electr Eng*. Published online 2014. doi:10.1016/j.compeleceng.2013.11.024

2. Lang M, Kotthaus H, Marwedel P, Weihs C, Rahnenführer J, Bischl B. Automatic model selection for high-dimensional survival analysis. *J Stat Comput Simul*. Published online 2015. doi:10.1080/00949655.2014.929131

3. Cox DR. Regression Models and Life-Tables. *J R Stat Soc Ser B*. Published online 1972. doi:10.1111/j.2517-6161.1972.tb00899.x

4. Ishwaran H. Variable importance in binary regression trees and forests. *Electron J Stat*. Published online 2007. doi:10.1214/07-EJS039

5. Tibshirani R, Saunders M, Rosset S, Zhu J, Knight K. Sparsity and smoothness via the fused lasso. *J R Stat Soc Ser B Stat Methodol*. Published online 2005. doi:10.1111/j.1467-9868.2005.00490.x

6. Tsamardinos I, Brown LE, Aliferis CF. The max-min hill-climbing Bayesian network structure learning algorithm. *Mach Learn*. Published online 2006. doi:10.1007/s10994-006-6889-7

7. Lagani V, Athineou G, Farcomeni A, Tsagris M, Tsamardinos I. Feature selection with the r package mxm: Discovering statistically equivalent feature subsets. *J Stat Softw*. Published online 2017. doi:10.18637/JSS.V080.I07

**APPENDIX 4.**

**Cox PH model**

The Cox PH model is considered the standard model for analyzing survival data^1^. The Cox PH model is semi-parametric (since the baseline hazard function, $h_{0}(t)$, is unspecified) and evaluates the effects of observed risk factors simultaneously on the time to an event of interest (e.g., diagnosis of a disease). It is the most frequently used method for modeling an individual’s survival, given their baseline data.

The Cox PH model is stated by the hazard function, which is the risk of an event occurring at time *t*. The formula for the Cox PH model is

$$h \left( t, X_{1},X_{2},\ldots X_{p} \right)=h_{0}\left( t \right)exp(\beta_{1}X_{1}+\beta_{2}X_{2}+\ldots+\beta_{p}X_{p})$$

where $h \left( t, X_{1},X_{2},\ldots X_{p} \right)$ is the expected or predicted hazard at time $t$ for a subject with covariate values, $X_{1},X_{2},\ldots X_{p}$, $h_{0}(t)$ is the baseline hazard when all the covariates equal to zero, $exp$ is the exponential function, $X_{i}$ is the $i^{th}$ covariate in the model, and $\beta_{i}$ is the regression coefficient for the $i^{th}$ covariate, $X_{i}$.

The Cox PH model does not assume a particular distribution for the survival times. The baseline hazard function is unspecified (no assumptions about the shape of the function), which can take any form and only a function of time (i.e., no covariates involved). However, the model is limited by some strict assumptions, such as the proportional hazards, and violation of these assumptions will result in entirely misleading results. The regression coefficients in the Cox PH model are estimated by maximizing the partial likelihood.

**Penalized Cox regressions (Lasso, Ridge, and EN)**

When applied to high-dimensional data (the number of features in the data is almost equal to or even exceeds the number of observations), the basic Cox model does not generalize well. The model may perform poorly and provide inaccurate results due to overfitting (when a model is tailored to a specific dataset and cannot generalize to other datasets)^2^. Overfitting can be prevented through regularization, a process of introducing additional information into the model. Several different penalty functions have been developed and introduced in prediction models to identify the most relevant features of the outcome in high-dimensional data. Such a model is called a penalized model, which adds penalty functions to restrict the features. This restriction reduces or shrinks the coefficient values toward zero to ensure that the model has less impact on the less relevant features.

The two most commonly used regularizers are the L1 and L2 penalties. In the L1 penalty (also known as Lasso), the sum of the coefficients’ absolute value is penalized, and feature selection and regression coefficient estimation are simultaneously performed. The L1 penalty yields sparse models (models with smaller features) that are more easily interpreted^3^.

The sum of squared coefficients is penalized in the L2 penalty (also known as Ridge regression). Unlike Lasso, Ridge regression cannot produce a sparse model, as any of the coefficients never become precisely zero, and hence none are eliminated. For the same reason, Ridge regression also cannot perform variable selection. Lasso suffers from limitations because it cannot select more features than the number of observations. In cases with correlated features, it tends to choose only one from a group without discrimination^4^. Lasso feature selection can be too data-dependent and therefore unstable.

EN emerged from Lasso’s criticism and provided a solution by combining the Ridge regression and Lasso penalties to get the best of both worlds. EN is a linear combination of the L1 and L2 penalties and can perform feature selection and deal with the correlation between the features simultaneously^4^. Unlike Lasso, EN can be useful when the number of features is larger than the number of observations.

**Random survival forest**

The random forest^5^ is an ensemble method specifically designed to make predictions using tree-structured models. RSF^6^ is an extension of the original Breiman’s random forest^5^ to censored survival data using a forest of survival trees for prediction. In an RSF, many bootstrap samples are randomly drawn from the given dataset, and for each sample, a survival tree is built by randomly selecting features. Each node is split based on randomly selected candidate features in an RSF to maximize the child nodes’ survival difference. Using the non-parametric Nelson-Aalen (NA) estimator, the ensemble Cumulative Hazard Function (CHF) of the bootstrapped samples is calculated by taking the CHF average of each tree^7^. Randomization in RSFs reduces the correlation among the trees and thus improves the predictive performance. RSF offers many advantages: the ability to model complex, nonlinear data, handle high-dimensional data, identify interactions, and naturally impute missing data, and it has become a popular and powerful tool for survival prediction^8^.

**Boosted gradient**

The idea behind boosting is to add new models to the ensemble sequentially. At each iteration, a new weak, base-learner model (where the error rate is only a little better than random guess) is trained concerning the error (residuals) of the whole ensemble learned so far and improved the remaining error iteratively. Once it reaches a stage where errors cannot be improved, the process can be stopped. Algorithmically, a loss function is minimized such that loss becomes its minimum. GB^9^ identifies the shortcomings of weak learners by using gradients in the loss function.

**REFERENCES**

1. Cox DR. Regression Models and Life-Tables. *J R Stat Soc Ser B*. Published online 1972. doi:10.1111/j.2517-6161.1972.tb00899.x

2. van Houwelingen HC, Putter H. *Dynamic Prediction in Clinical Survival Analysis*.; 2011. doi:10.1201/b11311

3. Hastie T, Tibshirani R, Friedman J. *Elements of Statistical Learning 2nd Ed.*; 2009.

4. Zou H, Hastie T. Regularization and variable selection via the elastic net. *J R Stat Soc Ser B Stat Methodol*. Published online 2005. doi:10.1111/j.1467-9868.2005.00503.x

5. Breiman L. Random forests. *Mach Learn*. Published online 2001. doi:10.1023/A:1010933404324

6. Ishwaran H, Kogalur UB, Blackstone EH, Lauer MS. Random survival forests. *Ann Appl Stat*. Published online 2008. doi:10.1214/08-AOAS169

7. Wang P, Li Y, Reddy CK. Machine Learning for Survival Analysis: A Survey. *arXiv*. Published online 2017.

8. Spooner A, Chen E, Sowmya A, et al. A comparison of machine learning methods for survival analysis of high-dimensional clinical data for dementia prediction. *Sci Rep*. Published online 2020. doi:10.1038/s41598-020-77220-w

9. Friedman JH. Stochastic gradient boosting. *Comput Stat Data Anal*. Published online 2002. doi:10.1016/S0167-9473(01)00065-2
